# 

**Published:** 1902-12-27

**Authors:** 


					the hospital. "The standard of highest purity."? Lancet. dec. 27th. 1902
% CADBURY'S -a i? CADBURY'S
COCOA HI Jrm COCOA
ABSOLUTELY PURE. )g?t C?   Z" ' NO DRUGS OR ALKALIES USED
LAsconi^M^fsffAJv UiFJUiAjut
No. 848. Yol. XXXIII. [fKpfpe"] Saturday,
Dec. 27th, 1902.
-SubacnptionTeraiB,] pRICH TWOPENCE.

				

